# A novel anti-EMMPRIN function-blocking antibody reduces T cell proliferation and neurotoxicity: relevance to multiple sclerosis

**DOI:** 10.1186/1742-2094-9-64

**Published:** 2012-04-05

**Authors:** Smriti M Agrawal, Claudia Silva, Janet Wang, Jade Pui-Wai Tong, V Wee Yong

**Affiliations:** 1 Departments of Clinical Neurosciences and Oncology, Hotchkiss Brain Institute and the, University of Calgary, 3330 Hospital Drive NW, Calgary, AB, T2N 4N1, Canada

**Keywords:** Experimental autoimmune encephalomyelitis, Extracellular matrix metalloproteinase inducer, Function-blocking antibody, Matrix metalloproteinases, Multiple sclerosis, Neuroinflammation T cell proliferation, Neurotoxicity

## Abstract

**Background:**

Extracellular matrix metalloproteinase inducer (EMMPRIN; CD147, basigin) is an inducer of the expression of several matrix metalloproteinases (MMPs). We reported previously that blocking EMMPRIN activity reduced neuroinflammation and severity of disease in an animal model of multiple sclerosis (MS), experimental autoimmune encephalomyelitis (EAE).

**Methods:**

To improve upon EMMPRIN blockade, and to help unravel the biological functions of EMMPRIN in inflammatory disorders, we have developed several anti-EMMPRIN monoclonal antibodies.

**Results:**

Of these monoclonal antibodies, a particular one, clone 10, was efficient in binding mouse and human cells using several methods of detection. The specificity of clone 10 was demonstrated by its lack of staining of EMMPRIN-null embryos compared to heterozygous and wild-type mouse samples. Functionally, human T cells activated with anti-CD3 and anti-CD28 elevated their expression of EMMPRIN and the treatment of these T cells with clone 10 resulted in decreased proliferation and matrix metalloproteinase- 9 (MMP-9) production. Activated human T cells were toxic to human neurons in culture and clone 10 pretreatment reduced T cell cytotoxicity correspondent with decrease of granzyme B levels within T cells. *In vivo*, EAE mice treated with clone 10 had a markedly reduced disease score compared to mice treated with IgM isotype control.

**Conclusions:**

We have produced a novel anti-EMMPRIN monoclonal antibody that blocks several aspects of T cell activity, thus highlighting the multiple roles of EMMPRIN in T cell biology. Moreover, clone 10 reduces EAE scores in mice compared to controls, and has activity on human cells, potentially allowing for the testing of anti-EMMPRIN treatment not only in EAE, but conceivably also in MS.

## Background

Extracellular matrix metalloproteinase inducer (EMMPRIN; basigin, CD147) is a member of the immunoglobulin (Ig) superfamily. It is a cell surface glycoprotein expressed on numerous cell types [1-4], with many binding partners including CD98, monocarboxylate transporters (MCT-1), cyclophilin, CD44, and hyaluronan to name but a few [5-7], and a long list of acronyms including tumor collagenase stimulatory factor (TCSF) [8]), M6 (in human cells) [3], neurothelin, 5A11 and HT7 (chicken) [9-11], OX47 and CE9 (rat) [1,12], and basigin and gp42 (human and mouse) [2,13]. EMMPRIN was most recently shown to induce the production of several matrix metalloproteinases (MMPs), resulting in its renaming to EMMPRIN for ‘Extracellular Matrix MetalloPRoteinase INducer’ [4,14].

EMMPRIN-null mice have abnormalities of body size, memory function [15], female reproduction, spermatogenesis [16,17], lung and liver tissue structure, blood–brain barrier integrity [18], T cell cycling in the thymus [19] and retinal function [20,21]. EMMPRIN-null mice are rarely born due to prenatal loss around the time of implantation; it is estimated that only about 30% of EMMPRIN-null embryos are born, of which 50% of surviving pups die of interstitial pneumonia within the first week of life [16]. Thus, investigating the biology of EMMPRIN via these constitutively null mice poses many challenges. We have explored the roles of EMMPRIN by the generation and use of anti-EMMPRIN function blocking antibodies in this study.

Multiple sclerosis (MS) is an immune-mediated disease of the central nervous system (CNS) with prominent demyelination and axonal degeneration. An animal model, experimental autoimmune encephalomyelitis (EAE), mimics several immunological and histological features of MS. Many leukocyte subsets infiltrate into the CNS in MS and EAE, and our group and others have previously reported on several MMPs crucial in this infiltration process [22-24]. We recently found an important role for EMMPRIN as an upstream regulator of the aberrant expression of MMPs in EAE [25]. In that report, we described that a commercially available function-blocking anti-mouse EMMPRIN antibody reduced MMP activity in the CNS resulting in lowered EAE clinical severity and decreased leukocyte infiltration into the CNS [25].

Although a number of anti-EMMPRIN antibodies are available commercially, these are generally used for particular applications and are species specific (Table 1). A monoclonal anti-EMMPRIN with multiple applications particularly function blocking activity, and capable of recognizing both murine and human cells, would be advantageous as a therapeutic application in neuroinflammatory disorders. In the present study, we describe our development of a novel anti-EMMPRIN function blocking monoclonal IgM antibody named clone 10 that detects and affects EMMPRIN activity on both murine and human cells. Our results suggest the potential use of clone 10 as a therapeutic antibody in MS and other neuroinflammatory disorders.

**Table 1:** Commercial antibodies to mouse or human extracellular matrix metalloproteinase inducer (EMMPRIN) utilized in this study

**Manufacturer (catalog no.)**	**Clone, species**	**Suggested use/dilution**
e-Bioscience (12-1471)	RL73.2, rat anti-mouse	Fluorescence-activated cell sorting (1:50)
Serotec (MCA2283)	OX-114, rat anti-mouse	Immunofluorescence (1:500)
R&D Systems (MAB972)	116318, mouse anti-human	Fluorescence-activated cell sorting (1:100)
US Biological (34-5600)	E2260-03, rabbit anti-human	Immunofluorescence (1:20-50)
Ancell (376-820)	UM-8D6, mouse anti-human	*In vitro* cell culture (10 μg/ml)

## Methods

### Generation of a function blocking monoclonal anti-EMMPRIN antibody

**Figure 1:**
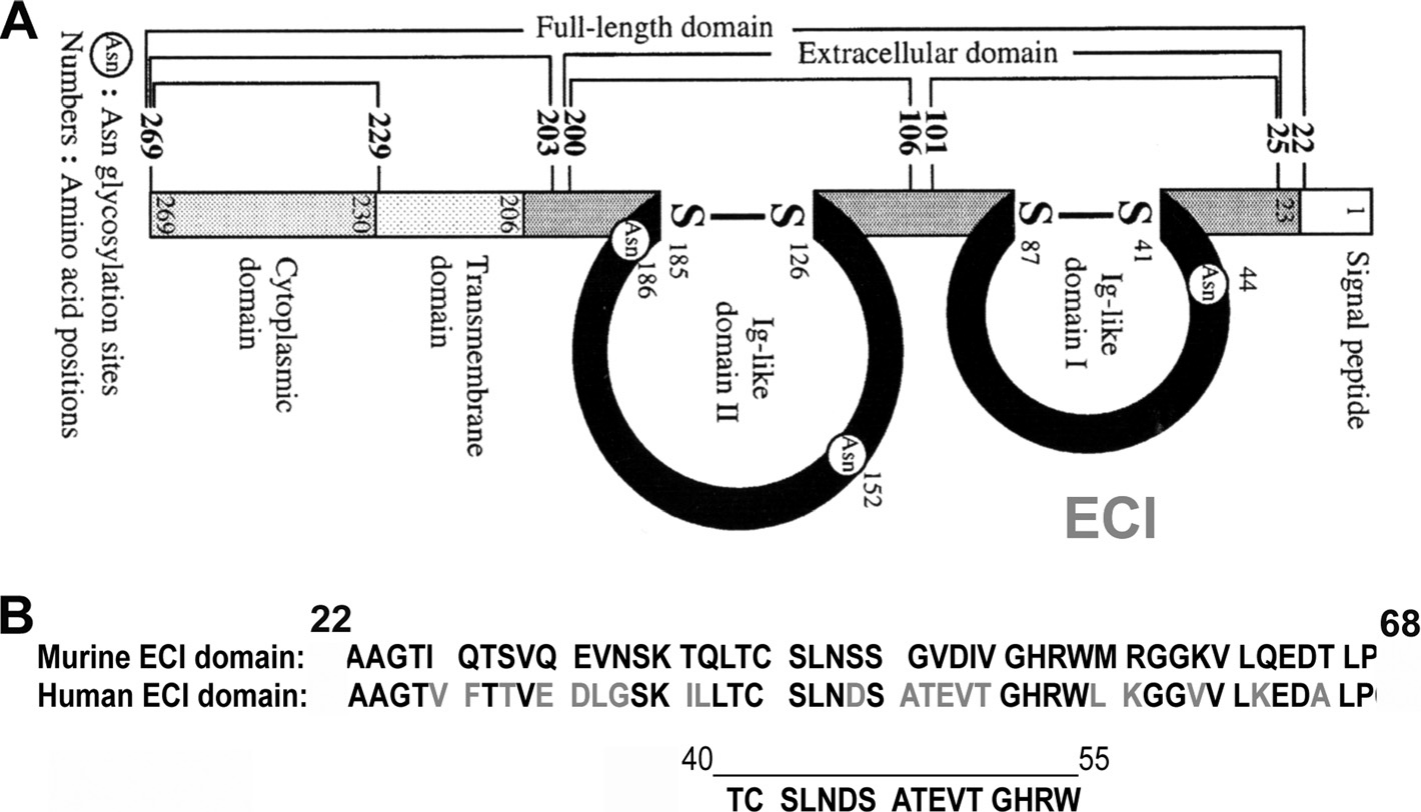
**Extracellular matrix metalloproteinase inducer (EMMPRIN) structure and peptide design.(A)** The schematic structure of human EMMPRIN shows that EMMPRIN consists of an extracellular domain, a transmembrane domain, a cytoplasmic domain and a signal peptide (based on [26]). The two extracellular domains (EC) contain immunoglobulin-like (Ig-like) domains. The numbers represent the positions of amino acid residues, and amino acids in circles in the two EC domains represent sites of glycosylation. **(B)** Amino acid sequence in murine EMMPRIN EC1 domain compared to human, with one peptide (40-55) selected based on the sequence responsible for the matrix metalloproteinase (MMP) induction function of EMMPRIN, and the region that has the most homology between mouse and human EMMPRIN.

The extracellular domain 1 (EC1; Figure 1) of EMMPRIN is responsible for the MMP induction function of EMMPRIN [26]. We identified the amino acid sequence in EC1 of murine and human EMMPRIN, determined sequences within EC1 with the highest homology between human and mouse EMMPRIN, and chose the peptide spanning residues 40 to 55 of human EMMPRIN (Figure 1) to be the immunogen. The University of Calgary’s Peptide Services generated this peptide. Briefly, BALB/C mice were subcutaneously immunized with the EMMPRIN peptide emulsified in Freund’s complete adjuvant. These mice were then boosted four times every 2 weeks with the EMMPRIN peptide emulsified in Freund’s incomplete adjuvant. ELISA was used to determine the serum titers of antibodies. At 3 days after the final booster injection, the mice were killed and spleens were removed. Hybridomas were produced by the cell fusion of Sp2/mIL6 mouse myeloid cells with spleen cells from the immunized mice. Hybridomas were grown and EMMPRIN-specific clones were selected by a positive reaction of hybridoma supernatant to the EMMPRIN peptide using ELISA, and verified using recombinant human EMMPRIN (R&D Systems, Minneapolis, MN, USA) and CNS tissue in western blots. Selected hybridoma clones were then inoculated into BALB/c mice by intraperitoneal injection. At 18 days after inoculation, ascitic fluid samples were harvested from mice and then centrifuged at 1,000 *g* to remove the solid fraction. IgM isotype of monoclonal antibodies against EMMPRIN from ascites were purified by Protein A-Sepharose CL-4B column chromatography.

### Western blots

Recombinant human EMMPRIN (rhEMMPRIN; 5 μg/ml) and mouse or human CNS tissues, sonicated in protein lysis buffer containing 1% Triton X-100 and protease inhibitor tablet (Roche Diagnostics, Mannheim, Germany), were separated by SDS-PAGE on 12% gels, transferred to polyvinylidene fluoride (PVDF) membranes, and probed using hybridoma supernatants from clones. Secondary anti-mouse antibodies conjugated with horseradish peroxidase (HRP) were added and detected using an ECL chemiluminesence kit (GE Lifesciences, Uppsala, Sweden). Membranes were imaged using a gel documentation system (Syngene, Frederick, MD, USA). EMMPRIN appears around 50 kDa in western blots [27].

### PCR for genotyping

Ear punch samples from pups 3 to 4 weeks old were digested in PBND digestion buffer (containing 50 mM potassium chloride; 10 mM Tris–HCl, pH 8.3; 2.5 mM magnesium chloride × 6 H_2_0; 0.1 mg/ml gelatin; 0.45% Nonidet P40; 0.45% Tween 20) with 20 μg/ml proteinase K, and incubated at 55°C, shaking at 550 rpm, overnight. Proteinase K was inactivated at 95°C for 10 minutes; samples were spun down and 1 to 2 μl of sample was used for PCR analysis. Using Taq polymerase and specific primers for EMMPRIN wild-type (reverse sequence: TGG CCT TCA CGC TCT TGA GC; forward: GCC TCA TCT CTA AGA TCA CT) and null (Neo1 R (neo1ATGATTGAACAAGATGGATTGCACG); Neo2 F (neo2 TTCGTCCAGATCATCCTGATCGAC)) in the presence of buffer, MgCl_2_, and deoxyribonucleotide triphosphates (dNTPs), the PCR reaction was carried out in a thermal cycler. Samples were run on a 2% agarose gel containing SYBR Safe DNA gel stain (Invitrogen, Carlsbad, CA, USA), and visualized using a Syngene Gel documentation system (Syngene, Frederick, MD, USA).

### Fluorescence-activated cell sorting (FACS)

Flow cytometry was performed using fluorescence-conjugated antibodies against CD45-PercP (leukocytes; BD Bioscience, Mississauga, Ontario, Canada), CD3-PE (T cells; BD Bioscience), CD14-PE (monocytoid cells; BD Bioscience), glial fibrillary acidic protein (GFAP)-fluorescein isothiocyanate (FITC) (astrocytes; Sigma, Oakville, Ontario, Canada), granzyme B-PE (T cell cytotoxicity; e-Bioscience, San Diego, CA, USA) or EMMPRIN-PE (CD147; e-Bioscience or R&D Systems). Briefly, cells were suspended in FACS buffer (FB; phosphate-buffered saline (PBS) + 2% fetal bovine serum), and blocked with an Fc blocker CD16/CD32 (1:100; BD Bioscience) for 20 minutes at 4°C. Cells were washed in FB and incubated in diluted antibodies of choice for 30 minutes at 4°C in the dark. Cells were washed in FB and fixed in 1% formalin before being resuspended in FB and analyzed using an LSRII FACS sorter.

### EMMPRIN-null embryos

EMMPRIN heterozygous breeding pairs, a kind gift from Dr Robert Senior (Washington University, St. Louis, MO, USA), were set up and females were monitored for plug formation to confirm mating. These heterozygote mice were originally generated by Dr Muramatsu [16] and given to Dr Senior for breeding and colony maintenance. We obtained permission from Dr Muramatsu for Dr Senior to transfer the mice to the University of Calgary animal facility. Plugged females were separated and 15 days later, these mice were humanely killed using ketamine/xylazine anesthetic solution. E15 embryos were individually dissected out and used in genotyping PCR, immunofluorescence staining and FACS analysis.

### Gelatin zymography

Gelatin gel zymography was used to identify MMP-2 and MMP-9 present in mouse CNS tissue as previously described [23]. Briefly, gelatin-binding proteins were first enriched by treatment of samples with gelatin sepharose, and they were then separated on a 10% polyacrylamide gel containing 1 mg/ml gelatin under non-reducing conditions. Gels were washed in a Triton X-100 renaturing buffer, followed by incubation in developing buffer containing 0.02% Brij 35, and then stained with Coomassie blue followed by destaining.

### Immunohistochemical and immunofluorescence staining

For immunofluorescence staining, CNS tissues from the UK MS tissue bank, or mouse E15 embryo tissues were sectioned using a cryostat (Lieca, Concord, Canada). Each tissue section mounted on a glass slide was fixed in -20°C methanol before blocking with 1% bovine serum albumin (BSA) in PBS for 30 minutes. Clone 10 (10 μg/ml), IgM isotype (10 μg/ml), or commercial anti-EMMPRIN antibody (10 μg/ml; Serotec; US Biological, Table 1) was applied to tissue sections and visualized using Alexa 488-conjugated anti-mouse secondary antibody (Jackson Laboratories, West Grove, PA, USA). Sections were examined and photographed using an Olympus BX51 fluorescence microscope and a Retiga 2000R camera (Q imaging, Surrey, BC, Canada). For immunohistochemical staining, mouse EAE or control spinal cords were snap frozen and sectioned using a cryostat (Leica, Concord, Canada). Tissue sections were mounted on a glass slide and dehydrated by dipping in increasing concentration of ethanol for 1 minute each, before being incubated in Luxol Fast Blue solution at 60°C for 1 h. Slides were then rehydrated in 95% and 70% ethanol before being dipped in 0.05% Lithium carbonate for 10 to 15 s and washed in distilled water. All sections were counterstained with hematoxylin and eosin and coverslips were applied using mounting media (Acrytol). Sections were viewed under an Olympus bright field microscope (BH2) equipped with an Olympus Q Color 3 digital camera. Images were acquired using Imagepro software (MediaCybernetics, Bethesda, MD, USA).

### Isolation and treatment of human T cells

Human peripheral blood mononuclear cells (PBMCs) were isolated from the blood of healthy adult volunteers by Ficoll-Hypaque centrifugation (GE Healthcare Life Sciences, Baie d’Urfe, Quebec, Canada) as previously described [28,29]. The PBMCs were washed once with phosphate-buffered saline (PBS) and suspended in serum-free AIM-V medium (Invitrogen Life Technologies, Burlington, Ontario, Canada). To activate T cells in the PBMC populations, 96-well round-bottomed plates were coated with 100 ng/ml of purified mouse anti-human CD3 (BD Pharmingen, Mississauga, Ontario, Canada) for a period of 3 h. Then 10 ng/ml of anti-CD28 (BD Pharmingen) was added as a suspension to anti-CD3 wells for further activation, and human PBMCs were plated at a density of 1 million cells/ml and cells were left for 3 days at 37°C in a 5% humidified CO2 incubator; we refer to the resultant anti-CD3/CD28 exposed cells as activated T cells. In some experiments, IgM isotype control (10 μg/ml; BD Bioscience), or clone 10 (10 μg/ml), or anti-EMMPRIN (10 μg/ml; Ancell, Bayport, MN, USA), or IgG isotype control (10 μg/ml; Ancell) were added. Certain PBMC preparations did not receive anti-CD3 or anti-CD28, and the floating cells collected 3 days thereafter are referred to as non-activated T cells.

### Carboxyfluorescein diacetate succinimidyl ester (CFSE) labeling

PBMCs were prepared at a concentration of 20 × 10^6^ cells/ml in incomplete RPMI media (containing 0.1% fetal bovine serum (FBS)). CFSE stocks (5 mM in dimethyl sulfoxide (DMSO); Molecular Probes, Eugene OR, USA) were diluted 1:40 in incomplete RPMI media, and 2 μl of this diluted CFSE solution was added per 0.1 ml of cells and mixed rapidly. After incubation at 37°C for 10 minutes, the cell solution was diluted with 12 ml of complete RPMI (containing 10% FBS) media, and incubated at room temperature for 5 minutes to quench the CFSE. The cells were then centrifuged twice and washed in complete RPMI media. Cells were counted and put into 96-well plates for treatment for 72 h. CFSE incorporation into cells and its dilution in proliferating cells was determined by FACS analysis.

### Human T cell and neuron cocultures

Brain from human fetuses of 16 to 20 weeks fetal age were obtained following therapeutic abortion according to guidelines approved by local institutional ethics committees. Neuronal cultures in excess of 90% purity were prepared as previously described [29,30]. Approximately 10 to 14 days after initial isolation, neurons were trypsinized from T75 flasks, resuspended in minimal essential medium (MEM) containing 10% fetal bovine serum, and seeded at 100,000 cells/well onto 96-well flat-bottomed plates precoated with polyornithine. Then, 2 days later, the medium was switched to AIM V medium and 100,000 PBMCs activated the previous 48 h with 100 ng/ml anti-CD3 and 10 ng/ml anti-CD28 were added to the neurons. After 24 h of coculture the plates were washed with PBS and fixed in 4% paraformaldehyde for 15 minutes, followed by incubation with mouse anti-microtubule associated protein-2 (MAP-2) antibody and secondary antibody to tag neurons. Hoechst dye was then applied to label all nuclei.

When clone 10 (10 μg/ml), or IgM isotype control (10 μg/ml; BD Bioscience), or commercial anti-EMMPRIN (10 μg/ml; Ancell, Table 1), or IgG isotype control (10 μg/ml; Ancell) were used to determine whether these alter T cell killing of neurons, they were added to PBMCs 3 h after the initiation of anti-CD3/CD28 exposure so that the initial activation of T cells was unimpaired. Antibodies were not re-added during the coculture of T cells and neurons so that their potential protective effect would be through activity of previously exposed T cells. To quantify the number of neurons remaining in wells as a function of neurotoxicity, images of MAP-2 and Hoechst-positive (4',6-diamidino-2-phenylindole (DAPI)) cells were captured using ImageXpress^MICRO^ (Molecular Devices, Sunnyvale, CA, USA), then quantified by MetaXpress^R^ Software (Molecular Devices) as per manufacturer instructions. Four sites were analyzed per well.

### Animals and EAE induction

Female C57BL/6 mice (Charles River, Wilmington, MA, USA) 6 to 8 weeks old were utilized for EAE immunization. All procedures are in accordance with guidelines of the Canadian Council of Animal Care and received approval from the local ethics committee. For immunization, 50 μg of myelin oligodendrocyte glycoprotein (MOG)_35-55_ peptide in complete Freund’s adjuvant containing 10 mg/ml of heat inactivated *Mycobacterium tuberculosis* H37RA (Difco, Lawrence, KS, USA) was injected subcutaneously, 50 μl on either side of the tail base. Animals were supplemented with 300 ng of pertussis toxin injected intraperitoneally on days 0 and 2 after MOG immunization. The animals were monitored daily for weight loss and changes of EAE disease score using a scale of 1 to 15 described previously [31].

### Clone 10 antibody administration to EAE mice

Clone 10 monoclonal antibody or rat IgM isotype control (BD Bioscience) was injected intraperitoneally (50 μg/mouse) in a volume of 100 μl into mice at days 8, 11 and 15 after MOG immunization.

### Statistical analysis

All results are expressed as mean ± SEM. The values were assessed for statistical significance using the SPSS V.19.0 software (SPSS, Chicago, IL, USA) to perform independent one-way analysis of variance (ANOVA) with Tukey *post hoc* comparisons, and differences were considered significant at *P* <0.01. For EAE experiments we performed a Mann–Whitney U non-parametric test and differences were considered significant at *P* <0.05.

## Results

### Western blot analyses suggest that clone 10 detects EMMPRIN in murine and human brain homogenates

**Figure 2:**
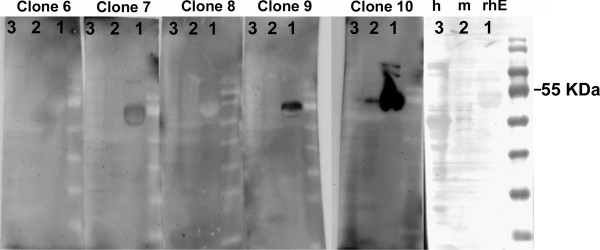
**Clone 10 binds most efficiently to extracellular matrix metalloproteinase inducer (EMMPRIN) in western blots.** Hybridoma supernatants from several clones are used in a western blot to detect 5 μg of recombinant human EMMPRIN (rhE; lane 1), 10 μg of mouse central nervous system (CNS) homogenate (m; lane 2) and 10 μg of human CNS homogenate (h; lane 3), EMMPRIN has an approximate molecular weight of 55 kDa.

In preliminary screenings, the conditioned media from several hybridoma clones were found to be immunoreactive to EMMPRIN peptide (40-55) (Figure 1) in ELISA assays (data not shown). We focused on the 12 clones (referred to as clones 1 to 13; no clone 5) with the strongest immunoreactivity and proceeded to western blots. We determined that clone 10 efficiently detected rhEMMPRIN and a similar 55 kDa band in mouse brain homogenates, while revealing a faint signal in human brain homogenate at the same molecular weight (Figure 2). The faint signal is likely due to a lower affinity of clone 10 for human EMMPRIN at the level of western blots, and/or to the low concentrations of anti-EMMPRIN antibody titer in the test hybridroma supernatants. Through isotyping analyses, clone 10 was found to be an IgM with a kappa light chain.

### Clone 10 efficiently and specifically binds EMMPRIN

**Figure 3:**
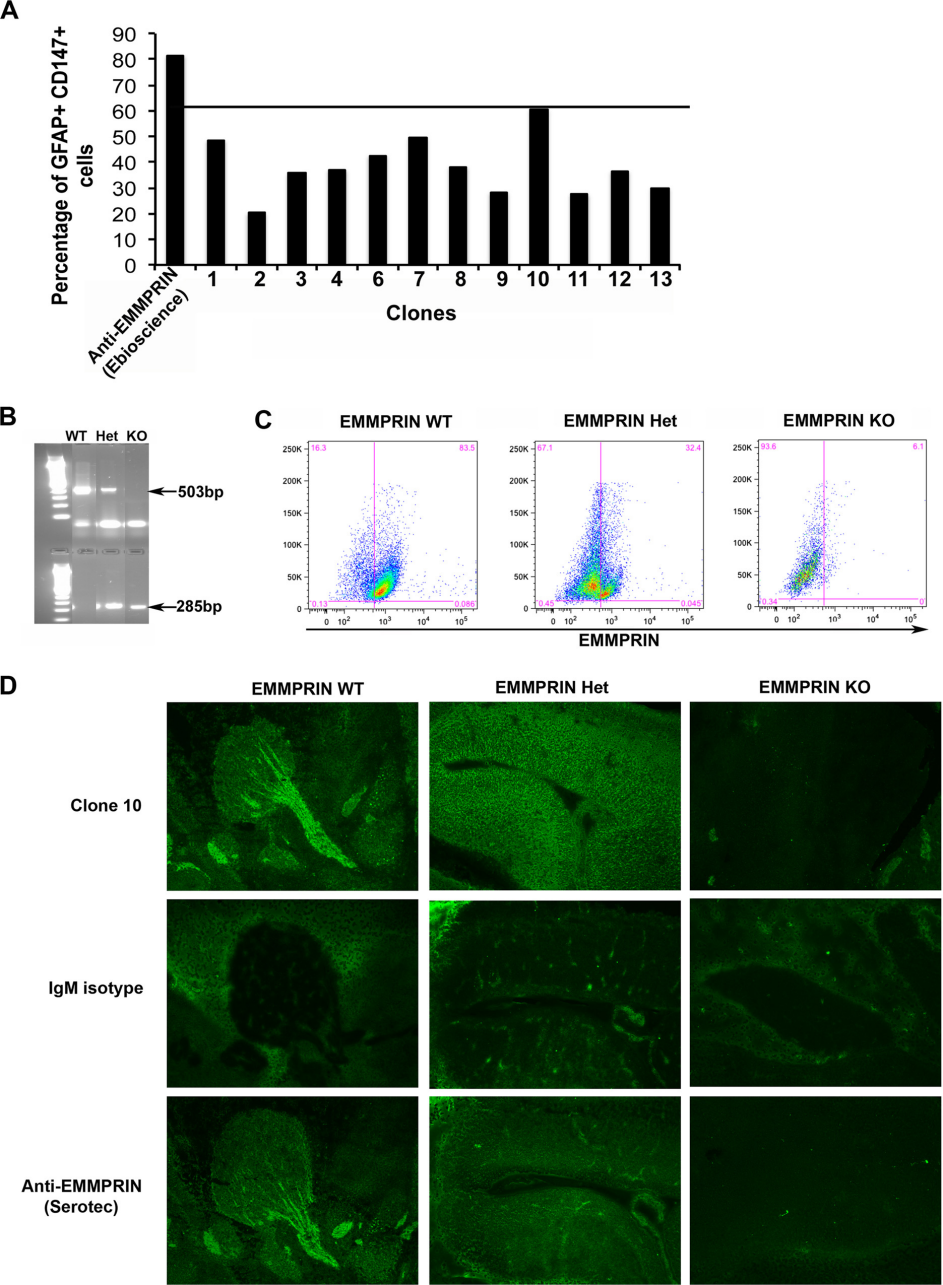
**Clone 10 specificity and binding efficiency in mouse tissue.** Using fluorescence-activated cell sorting (FACS) analysis **(A)**, mouse astrocytes identified by glial fibrillary acidic protein (GFAP) were examined for extracellular matrix metalloproteinase inducer (EMMPRIN) expression using hybridoma supernatants from several clones (1 to 13, no clone 5) and commercial anti-mouse EMMPRIN antibody (e-Bioscience). Tissues from E15 embryos were used to determine genotype by PCR **(B)**, where a band at 285 bp identified the PCR product for neo primers (knockout (KO) band), and a band at 503 bp identified the PCR product for EMMPRIN wild-type (WT); these tissues were subjected to FACS analysis **(C)** for EMMPRIN WT, heterozygous (Het) and KO mice. **(D)** EMMPRIN WT, Het and KO tissues were used in immunofluorescence staining with clone 10, IgM isotype control or commercial anti-EMMPRIN antibody (Anti-EMMPRIN; Serotec). Importantly, clone 10 and the Serotec antibody did not stain EMMPRIN KO tissue.

Astrocytes in the CNS are known to express EMMPRIN [25]. Thus, conditioned media from clones 1 to 13 (no clone 5) were used in FACS analyses on astrocytes from wild-type C57BL/6 mice. xWe found that the conditioned medium from clone 10 stained mouse astrocytes most efficiently compared to other clones, and that its efficiency (Figure 3A) approached that of a commercial anti-EMMPRIN antibody recommended for FACS (e-Bioscience, Table 1).

E15 embryos from pregnant mice from heterozygous mating were isolated and genotyped using PCR and FACS (Figure 3B,C). Sections from genotype-verified embryos were then stained with commercial anti-EMMPRIN antibody (Serotec, Table 1) and purified clone 10. Importantly, no staining was detected in EMMPRIN-null tissue using FACS or immunofluorescence with either antibody (Figure 3C,D), demonstrating that clone 10 antibody is specific for EMMPRIN.

### Clone 10 efficiently binds to human cells in culture and *in vivo*

**Figure 4:**
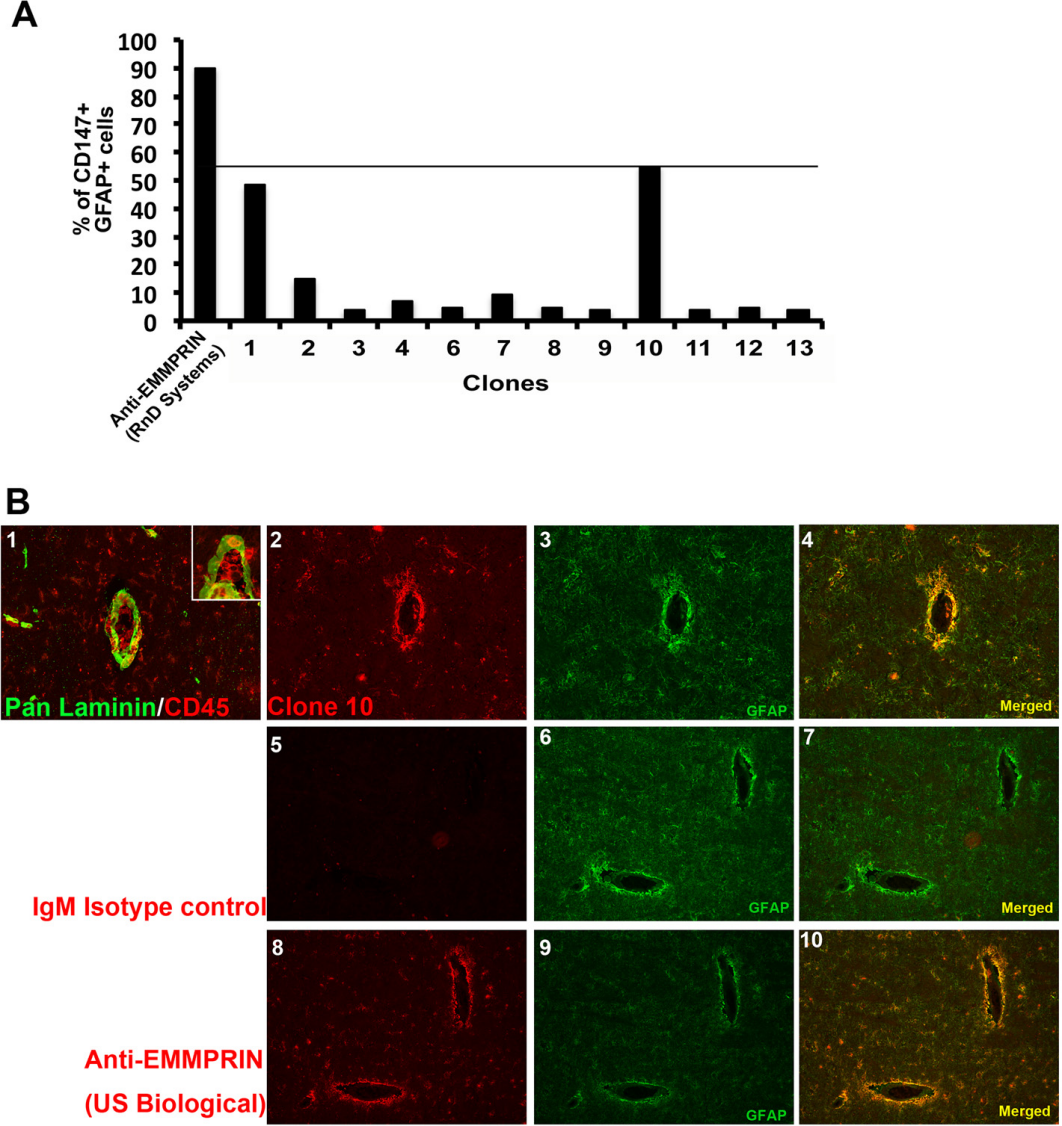
**Clone 10 binding efficiency in human cells and tissue.(A)** Using fluorescence-activated cell sorting (FACS) analysis, human fetal astrocytes stained for glial fibrillary acidic protein (GFAP) were additionally stained with hybridoma supernatants from several clones (1 to 13, no clone 5) and with the commercial anti-human extracellular matrix metalloproteinase inducer (EMMPRIN) antibody (R&D Systems). **(B)** Immunofluorescence staining of postmortem multiple sclerosis (MS) brain samples for laminin/CD45 (1; higher magnification inset) and GFAP (3, 4, 6, 7, 9, 10), and with clone 10 (2, 4), IgM isotype control (5, 7) and commercial anti-EMMPRIN (US Biological, Table 1) antibodies (8, 10).

We investigated whether clone 10 would bind to human cells given our goal of generating an anti-EMMPRIN antibody that could detect both mouse (Figure 3) and human cells. Thus, conditioned media from clones 1 to 13 (no clone 5) were used to stain human astrocytes in a FACS analysis (Figure 4A), and percentage of CD147+ (EMMPRIN) and GFAP + cells were compared to a commercial EMMPRIN antibody (R&D Systems, Table 1). Clone 10 stained human astrocytes most efficiently compared to the other clones, although clone 1 also stained astrocytes quite efficiently.

Next, we considered whether clone 10 could stain human brain sections. Given our previous report [25] of the upregulation of EMMPRIN in MS, we used tissue sections from MS specimens containing areas of perivascular inflammation (Figure 4B). Figure 4B1 shows immunoflourescence staining of postmortem human MS CNS samples with laminin and CD45 to illustrate a perivascular cuff found prominently in active MS cases. Laminin demarcates the basement membranes surrounding a post-capillary venule within the CNS and CD45 stains all infiltrating leukocytes. The presence of many leukocytes trapped within the laminin layers indicates a perivascular inflammatory cuff with ongoing immune cell influx into the CNS (inset of Figure 4B1). Staining for GFAP (glial fibrillary acidic protein; in green; Figure 4B, panels 3, 4, 6, 7, 9, 10) to detect astrocytes, and with clone 10 (Figure 4B2) and commercial anti-EMMPRIN (US biological, Table 1, Figure 4B8) shows a comparable expression of EMMPRIN (red staining) on astrocytes with both antibodies. Tissues stained with IgM isotype control (Figure 4B5) had minimal signal, implying staining specificity for EMMPRIN with clone 10.

### Clone 10 reduces proliferation of human T cells

**Figure 5:**
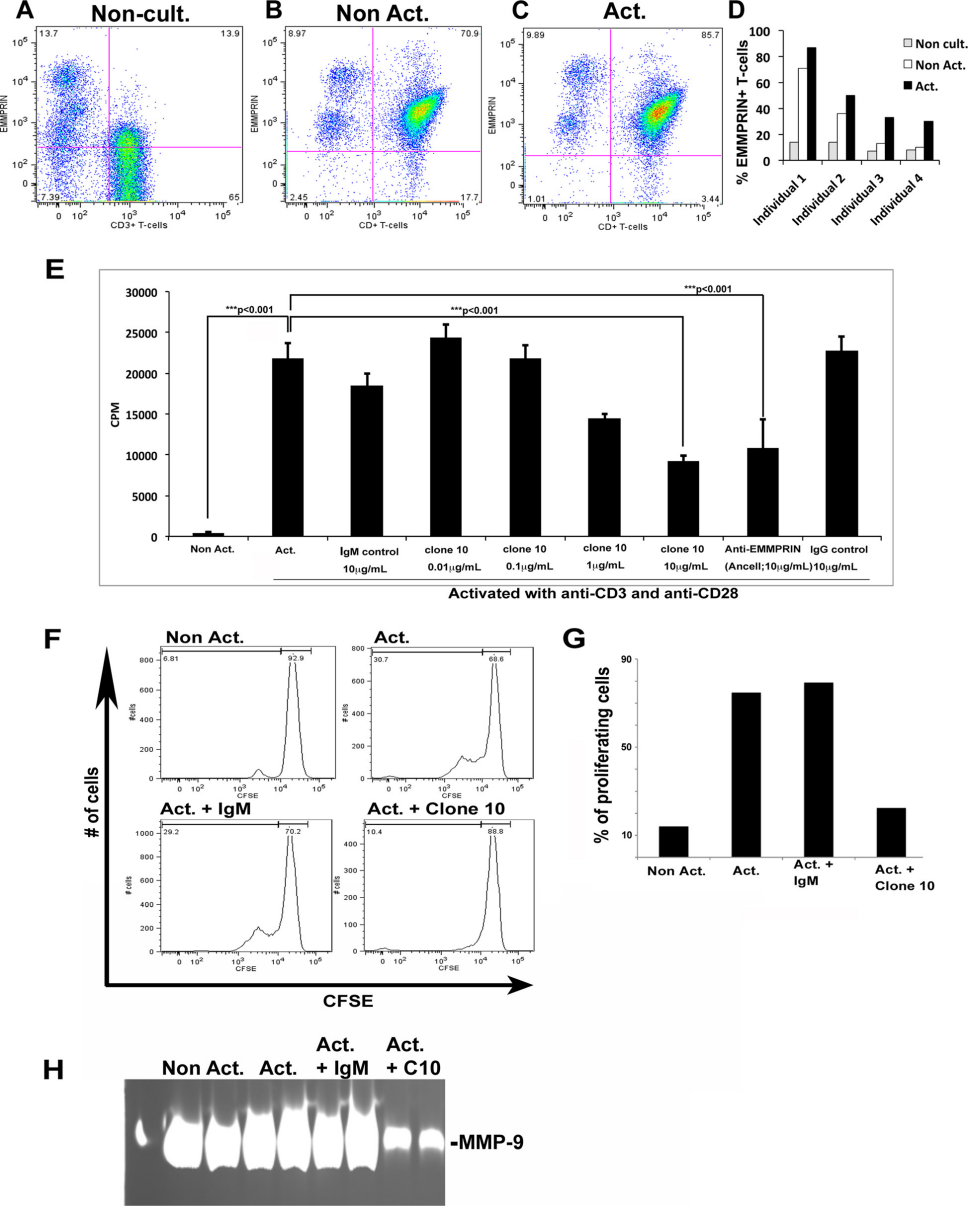
**Clone 10 treatment reduces T cell proliferation and activation.** Extracellular matrix metalloproteinase inducer (EMMPRIN) levels in non-cultured T cells immediately upon isolation (**(A)**; non-cult.), cultured without activation (**(B)**; non-act.), and cultured for 72 h with anti-CD3 and anti-CD28 (**(C)**; act.) are compared using fluorescence-activated cell sorting (FACS) analysis. EMMPRIN levels in T cells from four individual volunteers are displayed **(D)**. Human PBMCs incubated in the absence (non-act.) or presence (act.) of anti-CD3 and anti-CD28 were treated with various concentrations of clone 10, IgM isotype control, a commercial anti-human EMMPRIN antibody (Ancell) and IgG Isotype control. After 48 h cells were incubated with tritiated thymidine and proliferation assessed **(E)**; results are mean ± SEM of quadruplicates; ***P <0.001 compared to act. T cells (one-way analysis of variance (ANOVA) with Tukey’s *post hoc* comparisons). Similarly, non-activated and activated cells were treated with clone 10 or IgM isotype control and carboxyfluorescein diacetate succinimidyl ester (CFSE) intensity was determined in CD3+ cells by FACS analysis **(F)** and quantified **(G)**. Gelatin zymography was used to determine matrix metalloproteinase 9 (MMP-9) levels in conditioned media from peripheral blood mononuclear cells (PBMCs) non-activated (non-act.), activated (act.), activated and with clone 10 treatment (act. + C10), and activated with IgM isotype treatment (act. + IgM) **(H)**.

Non-cultured human T cells immediately post isolation express very low levels of EMMPRIN (non-cult.; Figure 5A,D). Non-activated human T cells that are cultured for 72 h express higher levels of EMMPRIN in comparison (non-act.; Figure 5B,D). Upon exposure to anti-CD3 and anti-CD28 to activate T cells, EMMPRIN expression increased in human PBMCs (act.; Figure 5C,D). These trends in EMMPRIN levels were similar in T cells from four individual volunteers (Figure 4D). To determine the roles of EMMPRIN, clone 10 and a commercial antibody to human EMMPRIN (Ancell; Table 1) were applied during the activation process with anti-CD3/CD28. The exposure to anti-CD3/CD28 significantly increased the proliferation of T cells as depicted by the increased incorporation of ^3^[H] thymidine (***P <0.001; Figure 5E), and the dilution of CFSE detected by FACS (act.; Figure 5F,G). Treatment of activated T cells with clone 10 or commercial anti-EMMPRIN antibody (Ancell; Table 1) significantly reduced T cell proliferation (***P <0.001; Figure 5E-G). This reduction in proliferation is EMMPRIN specific, as it was not observed when activated T cells were treated with the same amount of IgM isotype control (Figure 5E-G), or IgG isotype control (Figure 5E).

Furthermore, to examine a loss in the MMP-induction function of EMMPRIN by treatment with clone 10, we analyzed non-activated (non-act.) and activated (act.) T cells for their levels of MMP-9 using gelatin zymography. Conditioned media were examined since MMP-9 is a secreted protease. Figure 5H shows that activated T cells produce significant amounts of MMP-9 and this was reduced in clone 10 treated cells versus those treated with IgM isotype control (Figure 5H).

### T cell-mediated neuron killing

**Figure 6:**
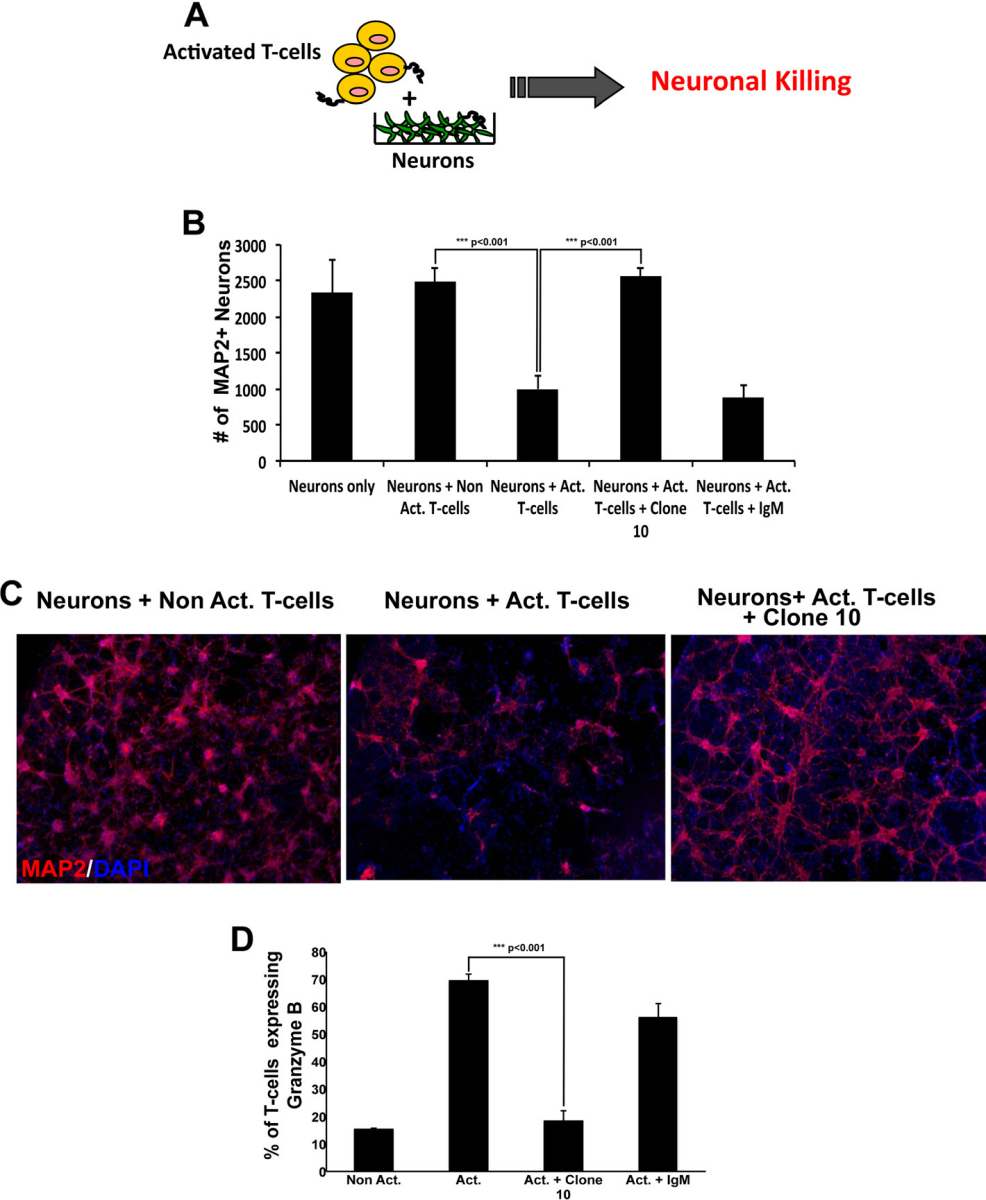
**Clone 10 treatment reduces T cell neurotoxicity.** Peripheral blood mononuclear cells (PBMCs) incubated in the absence (non-act.) or presence of anti-CD3 and anti-CD28 (act.) were applied to human fetal neurons, and neuronal killing was then assessed **(A)**. Using ImageXpress analysis, the number of microtubule-associated protein 2 (MAP2) + neurons in defined fields per well was enumerated; neuronal counts were reduced by activated T cells, representing neuronal death, and this was prevented by clone 10 treatment of T cells **(B)**. Values are mean ± SEM of quadruplicate wells; ***P <0.001 (one-way analysis of variance (ANOVA) with Tukey’s *post hoc* comparisons). **(C)** Representative images of MAP2-labeled neurons. Fluorescence-activated cell sorting (FACS) analysis was used to estimate Granzyme B levels in CD3+ T cells with or without treatment with clone 10 **(D)**. Values in (D) are mean ± SEM of triplicates samples; ***P <0.001 (one-way ANOVA with Tukey’s *post hoc* comparisons).

Activated T cells are toxic to human neurons in culture as previously described [29] (Figure 6). Using ImageXpress analysis, we found a significantly reduced number of microtubule-associated protein 2 (MAP2)-positive neurons in wells cocultured with activated T cells (neurons + act. T cells; ***P <0.001) but not in wells cocultured with non-activated T cells (neurons + non-act. T cells; Figure 6B,C). In the presence of clone 10, T cell mediated toxicity to neurons was significantly reduced, shown by more neurons in neuron-T cell cocultures (Figure 6B,C; ***P <0.001), compared to T cells treated with IgM isotype control (Figure 6B). Furthermore, granzyme-B levels, normally high in activated T cells and implicated as mediators of cytotoxicity [32] were significantly reduced in activated T cells treated with clone 10 compared to those treated with IgM isotype control (Figure 6D; ***P <0.001).

### Treatment with clone 10 reduces mouse EAE disease severity

**Figure 7:**
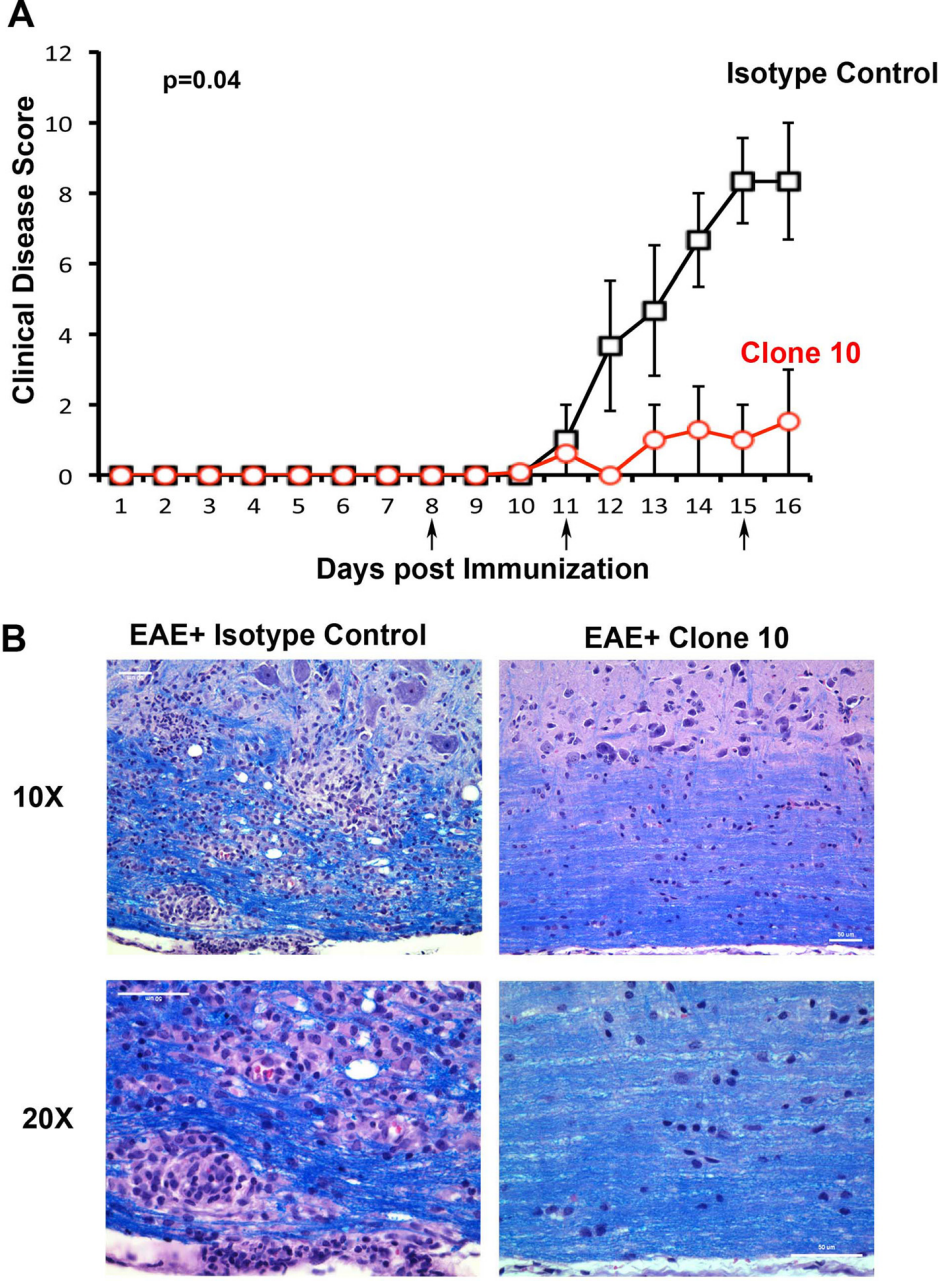
**Treatment with clone 10 attenuates experimental autoimmune encephalomyelitis (EAE) disease severity.(A)** Mice immunized for EAE were treated with 50 μg/mouse of clone 10 (red circles) or IgM isotype control (Black squares) at days 8, 11 and 15 (arrows) post-myelin oligodendrocyte glycoprotein (MOG) immunization. EAE disease severity was found to be significantly reduced in clone 10 treated animals compared with mice treated with an IgM isotype control. The data points have an overall statistical difference between timepoints 10 and 16 (*P = 0.04, Mann Whitney *U* test). Results are mean ± SEM from five mice each; this trend was reproduced in another experiment. **(B)** Spinal cord sections from EAE animals treated with clone 10 or IgM isotype control and killed at day 16 were stained with hematoxylin and eosin and Luxol Fast Blue to determine sites of immune cell infiltration and myelin sheaths. Images were captured at 10 × and 20 × original magnification.

We have previously demonstrated that [25] treatment of EAE mice around disease onset, with a commercially available anti-EMMPRIN antibody (e-Bioscience; Table 1), reduces EAE severity in these mice, compared to mice treated with an isotype control. In this study we wanted to examine whether our monoclonal anti-EMMPRIN antibody (clone 10) is capable of functioning similarly to the commercial anti-EMMPRIN antibody *in vivo*. We treated one group of EAE mice with clone 10 and another group with IgM isotype control on days 8, 11, and 15 post MOG immunization, and monitored EAE disease scores daily. EAE mice treated with clone 10 had significantly lower disease scores overall (*P* = 0.04; Mann–Whitney *U* test) compared to mice treated with IgM isotype control (Figure 7A). Histological analysis of longitudinal sections of spinal cords using hematoxylin and eosin with Luxol Fast Blue staining revealed spared myelin sheaths and the presence of little to no inflammatory cells in EAE animals treated with clone 10 compared to those treated with IgM isotype control (Figure 7B). These results are documented at both 10 × and 20 × original magnification to show differences between groups.

## Discussion

In MS and EAE, leukocytes cross the blood–brain barrier to enter the CNS parenchyma where they destroy myelin and the underlying axons [33]. Several roles for MMPs are implied in the pathology of MS and EAE [34-37], and many MMP members are elevated in the serum, cerebrospinal fluid and brain tissue of patients with MS (reviewed in [37]), and in the CNS of mice afflicted with EAE [22,31]. Importantly, MMPs are shown to be crucial in leukocyte trafficking into the CNS [38,39], specifically in the cleavage of components of the blood–brain barrier [23] that aids their entry into the CNS parenchyma.

The use of MMP inhibitors (reviewed in [37]) or using MMP-null mice [23,40-42] in EAE has proven to be challenging as several MMPs are upregulated simultaneously, and blocking one or two of them may be insufficient as many MMP members compensate for each other. To overcome this issue, we previously used a commercially available mouse specific anti-EMMPRIN function-blocking antibody (e-Bioscience) to treat EAE mice at various timepoints [25]. This treatment not only resulted in a marked decrease in MMP activity, but it reduced EAE disease severity. The utility of this commercial e-Bioscience antibody in EAE is not translatable to MS as the antibody has poor efficacy on human cells (data not shown).

The MMP induction function of EMMPRIN has been investigated quite thoroughly by several groups interested in tumor and cancer biology [4,8,43,44]. Specifically, the peptide sequence of EMMPRIN that is responsible for MMP induction has been identified [45] to lie within the EC1 (amino acid residues 22 to 50) domain in both mouse and human EMMPRIN. Interestingly, it is reported that the asparagine amino acid within this specific peptide region of EMMPRIN needs to be glycosylated for efficient MMP induction by EMMPRIN [46]. Many monoclonal antibodies for EMMPRIN have been reported to reduce MMP production by EMMPRIN in tumor cells both *in vitro* and *in vivo*[45,47-50], including the commercial anti-mouse EMMPRIN (e-Bioscience) we reported in EAE recently [25]. The biggest drawback of these antibodies is that they do not crossreact between mouse and human EMMPRIN.

To investigate the potential of an anti-EMMPRIN antibody therapy in MS, it would be desirable to have an antibody that will affect murine as well as human cells, so that preclinical work can be performed in mice prior to translation into humans. To develop an anti-EMMPRIN function-blocking antibody that crossreacts with mouse and human EMMPRIN, we chose a peptide spanning residues 40 to 55 of the MMP-inducing region of EC1 domain of EMMPRIN; this region also contains the site of glycosylation indicated to be important in efficient MMP induction by EMMPRIN [51], and it has maximum homology between mouse and human EMMPRIN (Figure 1).

In this study we describe a novel monoclonal function blocking anti-EMMPRIN antibody (clone 10) that recognizes both mouse and human targets in binding studies (Figures 3 and 4). Clone 10 also recognized rhEMMPRIN and a similar sized mass in mouse tissue in western blots (Figure 2), although it detected human tissue only faintly. This may be due to differentially glycosylated forms of EMMPRIN in human versus mouse samples that may mask EMMPRIN from clone 10 in human homogenates (Figure 2). Critically, we determined that clone 10 is specific for EMMPRIN by its staining of embryos that are EMMPRIN wild-type or heterozygote, but not in EMMPRIN-null embryos. Besides its capacity to bind both human and mouse tissue in western blots, clone 10 was useful in applications such as immunofluorescence staining and FACS analysis. These characteristics offer clone 10 as advantageous to other EMMPRIN antibodies that often are species specific, or applicable to a limited spectrum of uses. Moreover, clone 10 is a function-blocking antibody that reduced the proliferation of T cells, decreased the capability of T cells to secrete MMP-9 into the culture medium, and attenuated the cytotoxicity of activated T cells on neurons. Taken together, this information suggests clone 10 is a new and robust tool in understanding EMMPRIN biology and will help to unravel its roles in neuroinflammation and MS.

Human T cells that are activated with anti-CD3 and anti-CD28 proliferate and undergo cell cycling quite extensively. We find that treatment of activated T cells with clone 10 reduces their proliferative and cell cycling capacity (Figure 5). This finding is very interesting as it suggests a role for EMMPRIN in the T cell activation cascade, a function that may or may not be MMP dependent. Indeed, the latter is suggested by our finding that the proliferation of activated T cells is not altered by a pan-MMP inhibitor, BB94, at a concentration (100 nM) that prevents manifestation of MMP activity (data not shown). Thus, clone 10 may be a useful antibody to explore functions of EMMPRIN other than MMP induction.

Furthermore, it has been previously reported that activated T cells are cytotoxic [29,52] to human neurons with a role for a specific population of human T cells (CD4 + CD25 + CD127dimFoxp3+) [52] that use granzyme B as a mechanism to destroy neurons [32]. We show here that activated human T cells that are pretreated with clone 10 were less cytotoxic to human neurons compared to activated T cells that were not pretreated with any antibodies or those pretreated with IgM isotype control (Figure 6). These findings suggest a role for EMMPRIN in T cell cytotoxicity and provide further evidence that clone 10 is an effective function-blocking antibody. Specifically, our results suggest a role for EMMPRIN in the granzyme B mediated cytotoxic pathway of T cells.

Finally, to examine the therapeutic potential of clone 10 in MS, and to determine its effectiveness *in vivo*, we injected clone 10 into EAE animals just prior to onset, at onset and shortly after disease onset (Figure 7A). Compared to EAE animals treated with IgM isotype control, animals treated with clone 10 had lowered clinical disease scores (Figure 7A), and had more spared spinal cord tissue, with intact myelin sheaths and reduced inflammation (Figure 7B). These results suggest that clone 10 can potentially be used as an *in vivo* anti-EMMPRIN function-blocking antibody in future animal experiments prior to considering humanizing it for use in MS therapeutic trials.

## Conclusions

In this study we describe a novel monoclonal anti-EMMPRIN function-blocking antibody that crossreacts with mouse and human EMMPRIN, is specific for EMMPRIN, that not only efficiently blocks the MMP induction function of EMMPRIN, but also brings to light potential roles of EMMPRIN in neuroinflammatory diseases such as MS. Testing clone 10 in EAE demonstrated that blocking EMMPRIN is beneficial and suggests that the use of clone 10 could be a potential therapy for MS.

## Competing interests

The authors declare that they have no competing interests.

## Authors’ contributions

SMA mapped out areas to be used in the design of the EMMPRIN peptide to generate monoclonal antibodies, conducted the proliferation assays, performed the FACS analysis, EAE experiments, and stainings and drafted the manuscript. CS validated the monoclonal antibodies and maintained the hybridomas. JT and JW conducted the neuronal coculture experiments. VWY conceived the study, oversaw the experiments, and edited the manuscript. All authors read and approved the final manuscript.
